# Reciprocal Translocation T(Y;16) in a Male Patient With Non-obstructive Azoospermia: A Case Report and Literature Review

**DOI:** 10.7759/cureus.28365

**Published:** 2022-08-24

**Authors:** Yasser H Alharbi, Thamer M Alqurashi, Zohor A Azher, Abdulazziz S Baazeem

**Affiliations:** 1 Faculty of Medicine, Umm Al-Qura University, Makkah, SAU; 2 Department of Medical Genetics, Umm Al-Qura University, Makkah, SAU; 3 Department of General Surgery, Umm Al-Qura University, Makkah, SAU

**Keywords:** y and autosome chromosome translocation, azoospermia, infertility, andrology, urology

## Abstract

Translocation of sex/autosome chromosomes is uncommon, but they have a stronger impact on fertility than autosome/autosome translocation.^ ^Y/autosome translocation is associated with azoospermia in 80% of cases. To our knowledge, there have been only eight cases reported of a balanced reciprocal (Y;16) translocation associated with male infertility.Here we report an infertile man with azoospermia who has a reciprocal translocation t(Y;16) (q12; p13.2).

A 38-year-old Saudi medically free male presented with primary infertility and azoospermia for six years. He has a positive family history of male infertility. Physical examination was unremarkable. Investigations showed normal hormonal panel and azoospermia. He has a male karyotype with a reciprocal chromosome Y,16 translocation. Histopathology report of bilateral testicular sperm extraction (TESE) revealed most tubules show early maturation arrest and few show either Sertoli-cell only syndrome or are completely hyalinized and atrophic.

This case illustrates a rare cause of non-obstructive azoospermia in a male with chromosome Y,16 translocation as a result of a meiotic arrest. Medical practitioners should be aware of the genetic abnormalities of male patients who present with primary infertility. Karyotyping has the capability to diagnose genetic abnormalities in this patient.

## Introduction

Male infertility is a common condition. Nearly 50% of infertility cases are primarily caused by male factors [1]. Genetic abnormality is one of the leading causes of male infertility [2]. In azoospermic males, the rate of chromosomal rearrangement varies between 10% and 15%. Translocation of sex/autosome chromosomes is uncommon, but they have a stronger impact on fertility than autosome/autosome translocation [3]. In the general population, Y/autosome translocations occur in about 1 every 2000 people [4]. Y/autosome translocations may result in infertility or fertility depending on the Y chromosome breakpoint or the involved autosome [3]. Y/autosome translocation is associated with azoospermia in 80% of cases [5].

To our knowledge, there have been only eight cases reported of a balanced reciprocal (Y;16) translocation associated with male infertility. Here we report an infertile man with azoospermia who has a reciprocal translocation between the long arm of the Y chromosome and the short arm of the chromosome 16, t(Y;16)(q12;p13.2).

## Case presentation

**Figure 1:**
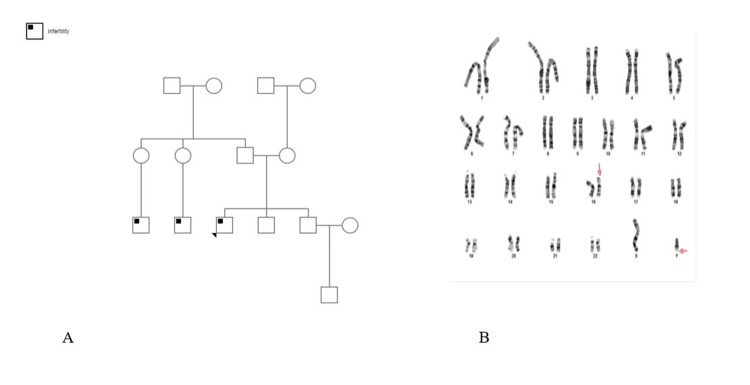
The family pedigree of fertility and the patient’s karyotype. A. The family pedigree of fertility. The arrow represents the patient. B. The patient’s karyotype. The arrows show the translocations between the Y chromosome and chromosome 16.

A 38-year-old Saudi male presented with primary infertility and azoospermia for six years. He was medically free, and his surgical history was significant for perianal fistula repair. No smoking history except for hookah pipe socially, he quit it six months ago. No other sources of gonadotoxin exposure were identified. His wife's age was 28 years, with no consanguinity. No contraception or assisted reproductive technologies (ART) were used. His family history showed unrelated parents. One of his brothers is married and has children, with no abortions or stillbirths. He has two paternal cousins who have had infertility for 10 years with unknown causes (Figure 1A). He has one maternal cousin who has Trisomy 21. He has no dysmorphic features were identified; he has normal secondary sexual characteristics. His genitalia examination revealed a normal phallus and normal scrotum with no clinical varicoceles. 

Investigations showed a normal hormonal panel (includes total testosterone, estradiol, prolactin, luteinizing hormone (LH), and follicle-stimulating hormone (FSH)), and several semen analyses were consistent with azoospermia that he performed previously. He has a male karyotype with a reciprocal translocation between the Y chromosome and chromosome 16 with breakage and reunion occurring at band Yq12 and 16p13.2. The segments distal to these bands were exchanged (Figure 1B). No Y chromosome micro-deletion was detected. 

Bilateral testicular sperm extraction (TESE) was done three years before the presentation and its histopathology report revealed that most tubules showed early maturation arrest and some (around 20%) showed Sertoli-cell only (SCO) syndrome on the right side. On the left, most tubules showed early maturation arrest, and few showed either SCO syndrome or are completely hyalinized and atrophic (around 10% each).

## Discussion

The most common abnormality linked with the loss or disruption of Yq chromosomal material is impaired spermatogenesis [6]. Fertile males are thought to have a breakpoint at Yq12, while infertile males have a breakpoint at the distal Yq11 euchromatic region, which includes the azoospermia factor (AZF) gene [3]. However, in addition to this case, we found some reports of infertility linked to Yq12 translocations and some reports with normal fertility who had breakpoints in the Yq11 euchromatic area [6].

In this case (Y;16) chromosome translocation can explain the nonobstructive azoospermia as a result of the meiotic arrest. Based on the clinical assessment, this is balanced translocation without other clinical consequences. The probability of finding sperms on retrieval is low. Considering the risk of this anomaly transmission and the risk of development of unbalanced chromosomal abnormalities in the offspring, preimplantation genetic diagnosis (PGD) with sex selection is recommended in case of successful retrieval. 

To our knowledge, there are eight cases of Y-chromosome 16 translocation with infertility. They have been summarized in Table 1. We found a case of a father who is a carrier of balanced Y;16 translocation t(Y;16) (q12;q22) and has apparently good fertility apart from no semen analysis results were included in the study. He has two healthy daughters and a son with unbalanced Y;16 translocation resulted in partial trisomy 16 and partial monosomy for the Y chromosome (46, X, der(Y)t(Y;16) (q12; q22)pat) that led to abnormal clinical consequences such as craniofacial anomalies and developmental delay [6], this explains the importance of PGD testing for patients with Y;16 translocation.

**Table 1:** Y-chromosome 16 translocation with infertility. ART: assisted reproductive technologies, NP: not present, ICSI: intracytoplasmic sperm injection, TESE: testicular sperm extraction, PGD: preimplantation genetic diagnosis

Number	References	Cases	Presentation	Semen Quality	ART	Comments
1	Not applicable	t(Y;16) (q12; p13.2)	Primary infertility	Azoospermia	NP	Current case.
2	Ghevaria H et al. 2017 [7]	46, X, t(Y;16) (q12;q13)	Primary infertility	Severe oligozoospermia	ICSI	This study aimed to present the meiotic outcome in embryos. The couple underwent six cycles of PGD.
3	Giltay J et al. 1999 [5]	reciprocal 46, X, t(Y;16) (q11.2l: q24)	Primary infertility	Severe oligoasthenoteratozoospermia (OAT)	ICSI	One chromosomally balanced child and two chromosomally normal children.
4	Giltay J et al. 1998 [8]	balanced translocation t(Y;16) (q11.21; q24)	Primary infertility	Oligozoospermia	ICSI	Healthy 46, XX child twin pregnancy, one with a 46, XX karyotype and the other a 46, X, t(Y;16) (q11.21; q24).
5	Jiang Y et al. 2012 [3]	Balanced 46, X, t(Y;16) (p11;q11)	Primary infertility	Azoospermia	NP	Retrieved sperms by right TESE.
6	Abeliovich D et al. 2009 [9]	46, X, t(Y;16) (q11,p13)	Primary infertility	Azoospermia	NP	Infertile brother with oligospermia 46XY- and fertile 4 brothers.
7	Gregori-Romero M et al. 1990 [10]	46, XY,t(Y;16)(q12;q11-12)	Primary infertility	Azoospermia	NP	This article is written in Spanish language.
8	Gunel M et al. 2008 [11]	balanced reciprocal translocation t(Y;16) (q12; q13)	Primary infertility	Azoospermia	NP	He had cryptorchidism, bilateral inguinal hernia repair with orchidopexy at the age of 8 years.
9	Faed M et al. 1982 [12]	46, X, t(Y;16) (q11;q13)	Partial block at spermatid formation Scanty sperm	Oligozoospermia	NP	Two times abortion.

## Conclusions

This case illustrates a rare cause of non-obstructive azoospermia in a male with chromosome Y,16 translocations as a result of a meiotic arrest. Medical practitioners should be aware of the genetic abnormalities of male patients who present with primary infertility. Karyotyping has the capability to diagnose genetic abnormalities in this patient.
